# Doping Ag in ZnO Nanorods to Improve the Performance of Related Enzymatic Glucose Sensors

**DOI:** 10.3390/s17102214

**Published:** 2017-09-27

**Authors:** Fan Zhou, Weixuan Jing, Pengcheng Liu, Dejun Han, Zhuangde Jiang, Zhengying Wei

**Affiliations:** State Key Laboratory for Manufacturing Systems Engineering, Xi’an Jiaotong University, Xi’an 710049, China; zhoufangreat@stu.xjtu.edu.cn (F.Z.); caterance@stu.xjtu.edu.cn (P.L.); handejun@stu.xjtu.edu.cn (D.H.); zdjiang@mail.xjtu.edu.cn (Z.J.); zywei@mail.xjtu.edu.cn (Z.W.)

**Keywords:** ZnO nanorod, doping, surface morphology, wettability, glucose sensor

## Abstract

In this paper, the performance of a zinc oxide (ZnO) nanorod-based enzymatic glucose sensor was enhanced with silver (Ag)-doped ZnO (ZnO-Ag) nanorods. The effect of the doped Ag on the surface morphologies, wettability, and electron transfer capability of the ZnO-Ag nanorods, as well as the catalytic character of glucose oxidase (GOx) and the performance of the glucose sensor was investigated. The results indicate that the doped Ag slightly weakens the surface roughness and hydrophilicity of the ZnO-Ag nanorods, but remarkably increases their electron transfer ability and enhances the catalytic character of GOx. Consequently, the combined effects of the above influencing factors lead to a notable improvement of the performance of the glucose sensor, that is, the sensitivity increases and the detection limit decreases. The optimal amount of the doped Ag is determined to be 2 mM, and the corresponding glucose sensor exhibits a sensitivity of 3.85 μA/(mM·cm^2^), detection limit of 1.5 μM, linear range of 1.5 × 10^−3^–6.5 mM, and Michaelis-Menten constant of 3.87 mM. Moreover, the glucose sensor shows excellent selectivity to urea, ascorbic acid, and uric acid, in addition to displaying good storage stability. These results demonstrate that ZnO-Ag nanorods are promising matrix materials for the construction of other enzymatic biosensors.

## 1. Introduction

Electrochemical enzymatic glucose sensors are of great importance in clinical diagnosis, biological analysis, food processing, and environmental monitoring domains [1,2]. Owing to their large surface-to-volume ratio, excellent electron transfer ability, stable chemical property, and good biocompatibility [3,4], different ZnO nanostructures are introduced on the working electrode of a glucose sensor to enlarge the surface area and increase the amount of immobilized GOx. Among them, one-dimensional ZnO nanostructures such as nanorods, nanotubes, nanocombs, and nanodisks, etc. [5,6,7,8] are much more utilized than ZnO nanolayers or nanoparticles due to their larger surface area and higher number of adsorption sites for more GOx [9]. Recent research has concentrated on the modification of these ZnO nanostructures to enhance the catalytic character or facilitate the electron transfer rate of glucose sensors [10,11].

The modification of ZnO nanostructures mainly includes depositing nanoparticles on the ZnO surface and doping nanoparticles in the ZnO crystal structure. Furthermore, these nanoparticles have good catalytic properties (such as Pt, Ni, and Co, etc.) [12,13,14] or excellent conductivity (such as Au, C, and Al, etc.) [15,16,17]. For example, Anusha coated Pt nanoparticles on ZnO nanoporous structures, and confirmed that the Pt nanoparticles enhance both the catalytic activity and electron transfer ability of the glucose sensor [12]. Chu hydrothermally doped Ni in ZnO nanorods, and concluded that doping with Ni improves the catalytic character of GOx towards glucose but decreases the conductivity of the ZnO nanorods [13]. Wei hydrothermally synthesized Au nanocrystals on the surface of ZnO nanorods, and demonstrated that the nanocrystals promote the direct electron transfer between GOx and ZnO nanorods [15]. Fidal doped Al in ZnO thin films, and proved that doping with Al increases the carrier concentration of the ZnO thin film as well as the sensitivity of the glucose sensor [17]. Obviously, these nanoparticles enhance the catalytic character of the glucose sensors, and more redox electrons are generated and transferred from the redox active centers of GOx to the ZnO surface. However, due to the limitation of the electron transfer ability of pristine ZnO, some electrons do not deliver rapidly from the ZnO to the electrode, and thus return back to the redox active centers of GOx, and ultimately have no contribution to the performance enhancement of the glucose sensor. Although an Al-doped ZnO thin film can collect these “escaped” electrons into the electrode effectively, Al is chemically active and the doping process is complex. Therefore, it is necessary to introduce other metals as dopants to improve the conductivity of ZnO.

Metal nanoparticles (such as Au and Pt) deposited on ZnO mainly promote direct electron transfer from the redox active centers of GOx to ZnO, but hardly facilitate the electron transfer from ZnO to the electrode. Though Au and Pt have higher work functions, few studies have reported doping with them in ZnO. On the other hand, Ag is a noble metal of good chemical stability and high electrical conductivity [18]. Moreover, Ag-doped ZnO is readily synthesized under facile conditions, and has been already applied in photo-degradation reactors and dye-sensitized solar cells to retard the recombination of photo-induced electron-hole pairs [19,20]. Hence, doping with Ag in ZnO has the potential to accelerate redox electrons transfer from ZnO to the electrode of a glucose sensor. Some studies have reported that doping metals in ZnO significantly influences the morphologies and wettability of the resultant ZnO nanostructures [21], affecting the area of the solid-liquid interface formed between electrode and analytic liquid, and ultimately the performance of the corresponding glucose sensor. However, the combined effects of the above influencing factors on the performance of glucose sensors still remain to be investigated.

In this paper, Ag-doped ZnO nanorods were hydrothermally synthesized on an indium tin oxide (ITO) electrode to improve the performance of a ZnO nanorod-based glucose sensor. Then the amounts of the doped Ag were regulated, and their influence on the surface morphologies, wettability, and electron transfer capability of the ZnO-Ag nanorods, as well as the catalytic character of GOx was studied. Further, the performance of the corresponding glucose sensor was optimized.

## 2. Materials and Methods

### 2.1. Materials and Reagents

The ITO electrodes (<7 ohm/sq) were bought from Zhuhai Kaivo Optoelectronic Technology Co., Ltd. (Zhuhai, China). Glucose oxidase (GOx, EC 1.1.3.4 from Aspergillus niger, ~200 U/mg), Nafion (5 wt %), d-(+)-glucose (99.5%), urea (U, 99.0–100.5%), uric acid (UA, ≥99.0%), and ascorbic acid (AA, >99.0%) were purchased from Sigma (St. Louis, MO, USA). Silver nitrate (AgNO_3_, 99.8%) was obtained from Aladdin (Shanghai, China). Acetone, absolute ethyl alcohol, zinc acetate dihydrate (Zn(CH_3_COO)_2_·2H_2_O, 99.9%), sodium hydrate (NaOH, 98.0%), zinc nitrate hexahydrate (Zn(NO_3_)_2_·6H_2_O, 99.9%), and hexamethylenetetramine (HMT, 99.0%) were purchased from Tianjin Kemiou Chemical Reagent Co., Ltd. (Tianjin, China). The phosphate buffer saline (PBS, 10 mM, pH 7.4) was prepared with Na_2_HPO_4_·12H_2_O and KH_2_PO_4_. The glucose solution (0.25 M) was kept for at least 24 h after preparation for mutarotation. All of the chemicals were analytical reagents and used without further purification. The water used was deionized water (R ≥ 18.2 MΩ·cm) produced by the Europtronic ultrapure water manufacturing system.

### 2.2. Hydrothermal Synthesis of ZnO-Ag Nanorods on the ITO Electrodes

The ITO electrodes were ultrasonically cleaned with acetone, absolute ethyl alcohol, and deionized water sequentially and dried in nitrogen flow. ZnO seed solution was prepared with 3 mmol Zn(CH_3_COO)_2_·2H_2_O and 6 mmol NaOH mixed in 100 mL absolute ethyl alcohol. The ITO electrodes were immersed in the ZnO seed solution for 1 min, dried at room temperature, and then annealed at 120 °C for 10 min to make a homogeneous crystal nucleus layer.

For the hydrothermal synthesis of ZnO-Ag nanorods, 5 mmol Zn(NO_3_)_2_·6H_2_O and 5mmol HMT were dissolved into 100 mL deionized water, followed by the addition of different amounts of AgNO_3_ (0, 1, 2, and 3 mM) as the Ag^+^ source. Then, the growth solution was stirred at 1000 rpm and heated to 90 °C. Subsequently, ZnO and ZnO-Ag nanorods were synthesized by maintaining the ITO electrodes with a ZnO crystal nucleus in the growth solution at 90 °C for 10 h. Finally, all the samples were rinsed with deionized water and dried at room temperature, thus the ZnO and ZnO-Ag nanorods-modified ITO (ZnO/ITO, ZnO-Ag/ITO) electrodes were obtained. To be convenient, the ZnO-Ag nanorods with doped Ag of 1, 2, and 3 mM are designated as ZnO-Ag-1, ZnO-Ag-2, and ZnO-Ag-3 nanorods, and their corresponding electrodes are referred to ZnO-Ag-1/ITO, ZnO-Ag-2/ITO, and ZnO-Ag-3/ITO electrodes in the following.

### 2.3. Immobilization of GOx on the ZnO/ITO and ZnO-Ag/ITO Electrodes

Prior to the immobilization of GOx, the ZnO/ITO and ZnO-Ag/ITO electrodes were rinsed with PBS, and then freshly prepared GOx solution (10 μL, 40mg/mL) was dropped on them. After water evaporated at 4 °C, 5 μL Nafion was coated on the electrodes, forming ion transparent membranes to prevent the leakage of GOx. Thus, the Nafion/GOx/ZnO/ITO, Nafion/GOx/ZnO-Ag-1/ITO, Nafion/GOx/ZnO-Ag-2/ITO, and Nafion/GOx/ZnO-Ag-3/ITO glucose enzymatic electrodes were constructed. When not in use, the electrodes were preserved at 4 °C.

### 2.4. Characterization of the ZnO and ZnO-Ag Nanorods

The morphologies of the ZnO and ZnO-Ag nanorods were characterized by field emission scanning electron microscopy (FE-SEM, SU8000, Hitachi, Tokyo, Japan). The crystal structures of the ZnO and ZnO-Ag nanorods were examined with an X-ray diffractometer (XRD, Panak, Almelo, Netherlands) using CuKα radiation, which was generated at a voltage of 40 kV and a current of 40 mA with the diffraction angle of 20–80°. The contact angles (CAs) for each sample were measured at five different regions with a contact angle analyzer (OCA20, Dataphysics, Stuttgart, Germany), and the values were averaged. The profiles of the ZnO/ITO and ZnO-Ag/ITO electrodes were extracted by combining FE-SEM images with the Image Processing Toolbox of MATLAB, and then the least square circles were fitted. With the least square circles subtracted from the extracted profiles, the surface heights of the ZnO/ITO and ZnO-Ag/ITO electrodes were obtained. According to the surface heights, the characteristic parameters including roughness Ra, skewness Sk, kurtosis Ku, and correlation length ξ were determined, and the surface morphologies of the ZnO and ZnO-Ag nanorods were characterized. The detailed calculation of the characteristic parameters is available in our previous work [22].

### 2.5. Electrochemical Measurements of the Glucose Sensors

All the measurements were performed on an electrochemical workstation (CHI660D, Shanghai Chenhua Instrument Co., Ltd., Shanghai, China) with the three-electrode system, which includes the prepared electrode as the working electrode, a Pt wire as the auxiliary electrode, and an Ag/AgCl with saturated KCl solution as the reference electrode. The electron transfer properties between the electrode surface and electrolyte were investigated with electrochemical impedance spectroscopy in PBS. The frequency ranged from 0.01 Hz to 100 kHz and the bias potential was +0.1 V. The electrochemical dynamic properties of the glucose sensors were studied by cyclic voltammetry in 3 mM glucose solution with a potential of −0.2 V–+0.8 V and a scan rate of 50 mV/s. The performance of the glucose sensors was evaluated by amperometric response. With the background current decaying to the steady state, 100 μL glucose solution was successively added in 50 mL PBS until the response current approximately became saturated. The applied potential was +0.8 V whilst the solution was being stirred at 400 rpm. All the applied potentials were with respect to the reference electrode.

## 3. Results and Discussion

### 3.1. Characterization of the ZnO and ZnO-Ag Nanorods

**Figure 1:**
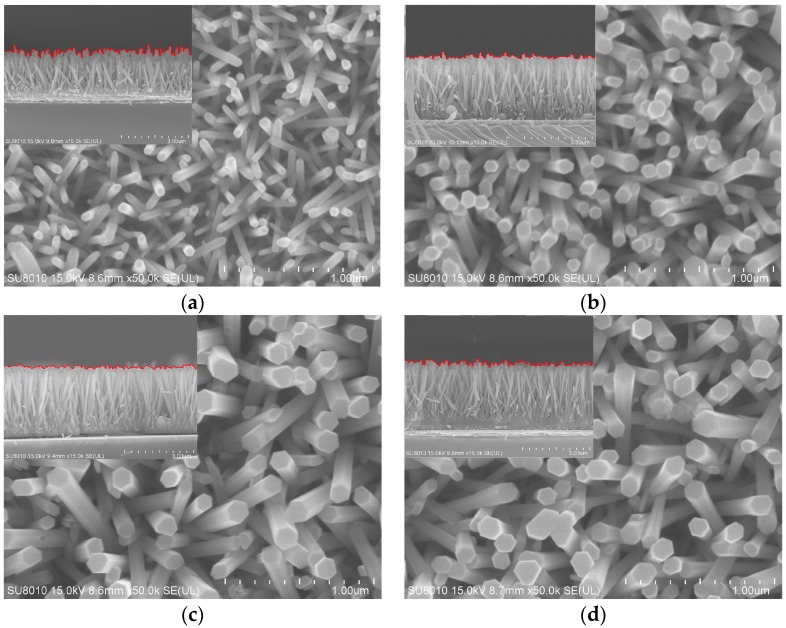
The FE-SEM images of the ZnO (**a**), ZnO-Ag-1 (**b**), ZnO-Ag-2 (**c**), and ZnO-Ag-3 (**d**) nanorods. The top left insets are the corresponding cross-sections.

Figure 1a–d indicate that both the ZnO and ZnO-Ag nanorods are hexagonal structures and grow vertically on the ITO substrates. In comparison to the ZnO nanorods, the ZnO-Ag nanorods are less random and more vertical to the ITO substrate, and both the diameters and lengths increase, see the insets of Figure 1a–d. When AgNO_3_ is added to ZnO growth solution, Ag^+^ occupies the position of some Zn^2+^, and the radius of Ag^+^ (1.26 Å) is larger than that of Zn^2+^ (0.74 Å), thus the diameters and lengths of the ZnO-Ag nanorods increase.

**Figure 2:**
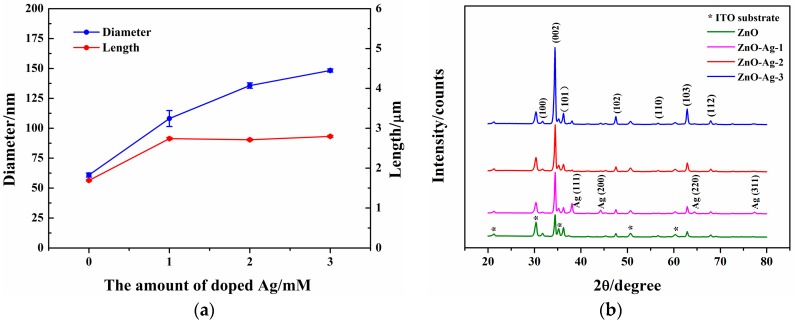
(**a**) The diameters and lengths of the ZnO and ZnO-Ag nanorods; (**b**) XRD patterns of the ZnO and ZnO-Ag nanorods.

Figure 2a shows that the diameters of the ZnO-Ag nanorods increase remarkably and the lengths almost remain unchanged when the amount of doped Ag increases. The XRD patterns in Figure 2b demonstrate that all of the diffraction peaks except those of Ag and the ITO substrate are indexed as hexagonal wurtzite ZnO (JCPDS card file No. 36-1451). No other diffraction peaks are found, suggesting the good purity of the ZnO and ZnO-Ag nanorods. The intensity of (002) diffraction peaks of the ZnO-Ag nanorods are much stronger than that of the ZnO nanorods. This implies that the doped Ag facilitates the growth of ZnO nanorods along the [001] crystal orientation. Therefore, the ZnO-Ag nanorods are arranged in a more orderly fashion and are more vertical to the ITO substrate. The results are consistent with the conclusions drawn from Figure 1a–d.

### 3.2. Surface Morphologies and Wettability of the ZnO and ZnO-Ag Nanorods

**Figure 3:**
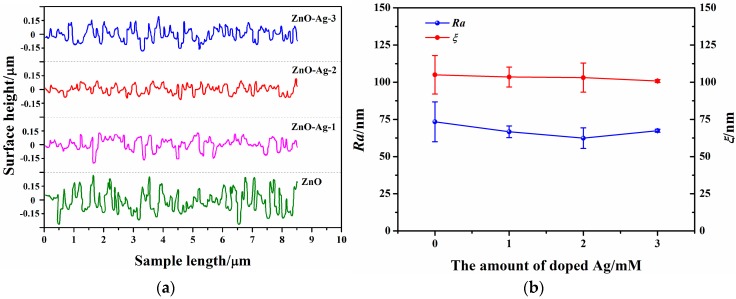

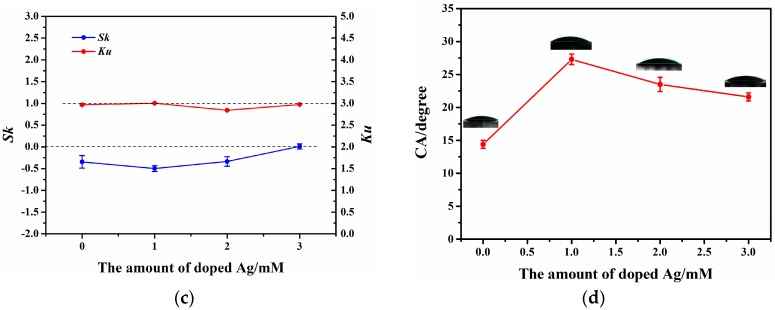
(**a**) Surface heights of the ZnO/ITO and ZnO-Ag/ITO electrodes. Characteristic parameters Ra, ξ; (**b**) and Sk, Ku (**c**) of the surface morphologies of the ZnO and ZnO-Ag nanorods; (**d**) Wettability of the ZnO and ZnO-Ag nanorods.

Figure 3a demonstrates that the surface heights of the ZnO-Ag/ITO electrodes fluctuate slightly (especially for the ZnO-Ag-2/ITO electrode) in comparison to that of the ZnO/ITO electrode. It turns out that doping with Ag again enhances the orientation uniformity of the ZnO-Ag nanorods and weakens their surface randomness. As a result, Ra becomes smaller and reaches the minimum value with doped Ag of 2 mM, and ξ nearly remains constant, referring to Figure 3b. This suggests that the surfaces of the ZnO-Ag nanorods become smoother. Figure 3c implies that as the amount of the doped Ag increases, Sk approaches from negative values to zero, whilst Ku stays around 3 except for that at 2 mM. This indicates that the surface heights of the ZnO-Ag nanorods exhibit a narrower distribution range. The changes of the surface morphologies demonstrate that doping with Ag does not benefit the hydrophilicity of the ZnO nanorods.

Figure 3d shows that the CAs of the ZnO-Ag nanorods increase compared with that of the ZnO nanorods. This agrees well with the fact that the CA of a hydrophilic surface enlarges when Ra decreases [23]. This shows that doping with Ag in ZnO nanorods diminishes the contact area of the solid-liquid interface between the electrode and analytic liquid, which is not good for the performance enhancement of the glucose sensor. The surface energy of the ZnO and ZnO-Ag nanorods is estimated to be 71.7, 68.8, 69.8, and 70.2 mJ/m^2^ [24]. Therefore, doping with Ag weakens the adsorption strength between GOx and the ZnO-Ag nanorods [25].

### 3.3. Electrochemical Properties of the Glucose Sensors

#### 3.3.1. Electrochemical Impedance Spectroscopy

**Figure 4:**
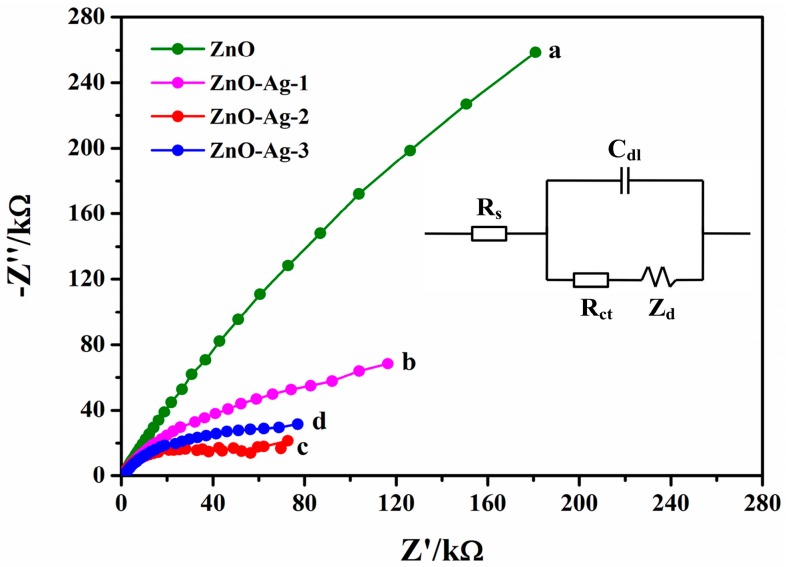
Electrochemical impedance spectroscopy of the Nafion/GOx/ZnO/ITO and Nafion/GOx/ZnO-Ag/ITO glucose sensors in phosphate buffer saline (PBS). The frequency ranges from 0.01 Hz to 100 kHz at a bias potential of 0.1 V vs. Ag/AgCl. The inset is the Randle equivalent circuit.

Figure 4 shows the electrochemical impedance spectroscopy of the Nafion/GOx/ZnO/ITO and Nafion/GOx/ZnO-Ag/ITO glucose sensors. The inset exhibits the Randle equivalent circuit, where R_s_ represents the electrolyte solution resistance, R_ct_ the charge transfer resistance, Z_d_ the Warburg impedance, and C_dl_ the double layer capacitance. The values of R_ct_ equal to the radii of the curves a-d, and are calculated to be 854.1, 147.5, 51.6 and 73.1 kΩ. R_ct_(b), R_ct_(c), and R_ct_(d) are much smaller than R_ct_(a), suggesting that the doped Ag remarkably facilitates the electron transfer ability of the ZnO-Ag nanorods. Therefore, R_ct_ decreases and the electron transfer ability of the ZnO-Ag nanorods enhances as the amount of doped Ag increases. However, R_ct_(c) is smaller than R_ct_(d). This might be ascribed to the formation of Ag_2_O on the ZnO surface. This shows that excessive Ag is not favorable to the electron transfer of the ZnO-Ag nanorods.

#### 3.3.2. Cyclic Voltammogram

**Figure 5:**
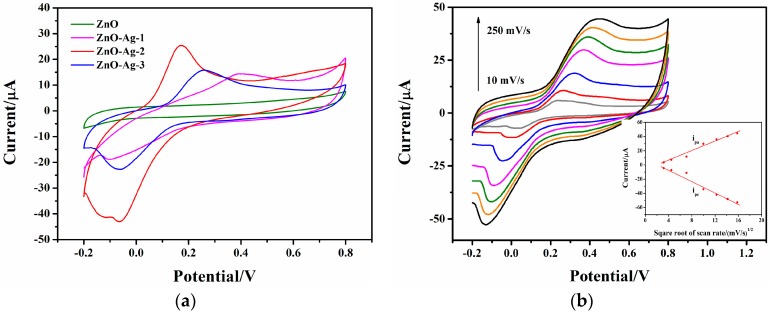
(**a**) Cyclic voltammograms of the Nafion/GOx/ZnO/ITO and Nafion/GOx/ZnO-Ag/ITO glucose sensors in 3 mM glucose solution. The voltage ranges from −0.2 to +0.8 V at a scan rate of 50 mV/s; (**b**) Cyclic voltammograms of the Nafion/GOx/ZnO-Ag-2/ITO glucose sensor at scan rates of 10, 20, 50, 100, 150, 200, and 250 mV/s. The inset shows the plots of the anodic and cathodic peak currents vs the square root of the scan rate.

Figure 5a indicates that no redox peaks appear for the Nafion/GOx/ZnO/ITO glucose sensor. In contrast, a pair of well-defined redox peaks occurs and the redox currents enlarge for the Nafion/GOx/ZnO-Ag/ITO glucose sensors. Moreover, the oxidation peak potential diminishes and the oxidation current rises with increasing amounts of doped Ag. This reveals that the catalytic activity of GOx is improved. However, the oxidation peak potential becomes larger and the oxidation current smaller when the amount of the doped Ag is 3 mM. This might also be attributed to the formation of Ag_2_O on the ZnO surface.

Figure 5b exhibits the effect of scan rate on the cyclic voltammetric performance of the Nafion/GOx/ZnO-Ag-2/ITO electrode. Both the oxidation and reduction peak currents increase as the scan rate ranges from 10 to 250 mV/s. For a diffusion-limited process, the relationship between peak current and scan rate is shown as $i_{p}=2.69\times 10^{5}n^{3/2}D^{1/2}\nu^{1/2}\mathrm{Ac}$ [26], where n is the number of exchanged electrons, D the diffusion coefficient of reactant, ν the scan rate, A the effective area of the electrode, and c the concentration of the reactant. Apparently when the scan rate rises, the peak current increases and is proportional to the square root of the scan rate. In this work, the peak current is also linear to the square root of the scan rate (see inset). This indicates that a diffusion-controlled electrochemical process occurs on the Nafion/GOx/ZnO-Ag-2/ITO electrode. Therefore, the electrochemical reaction is mainly controlled by the diffusion process and analyte diffusion occupies a great deal of reaction time. Consequently, the peak current scarcely has anything to do with the time taken by GOx in enhancing the rate of redox reactions.

Although doping with Ag in ZnO nanorods slightly smoothes the surface and weakens the hydrophilicity of the ZnO-Ag nanorods, in addition to diminishing the contact area of the solid-liquid interface (refer to Section 3.2), it remarkably improves both the electron transfer ability of the ZnO-Ag nanorods and the catalytic character of GOx. Hence, the performance of the corresponding glucose sensors is expected to be enhanced.

#### 3.3.3. Amperometric Response

**Figure 6:**
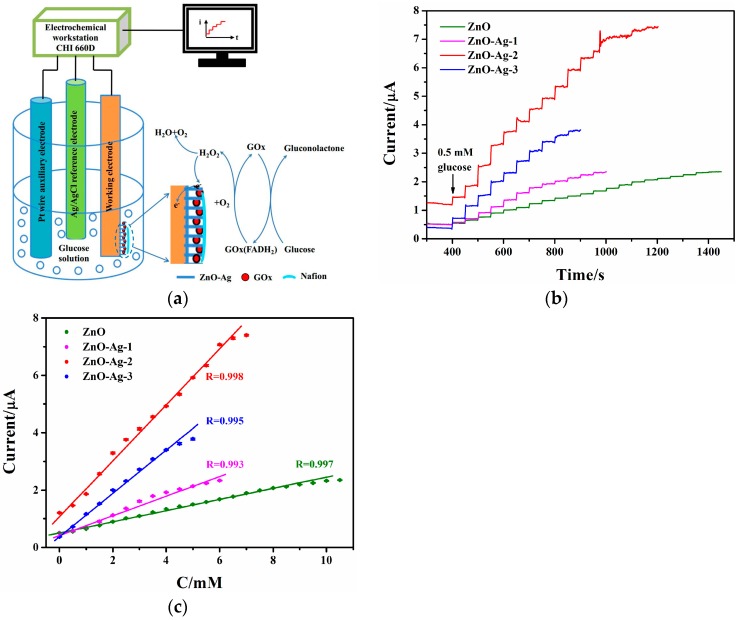
(**a**) The schematic diagram of the three-electrode system and the reaction on the working electrode; (**b**) Amperometric response of the Nafion/GOx/ZnO/ITO and Nafion/GOx/ZnO-Ag/ITO glucose sensors with the successive addition of 100 μL glucose solution in 50 mL PBS at +0.8 V; (**c**) The calibration curves of response current vs glucose concentration.

Figure 6a exhibits the schematic diagram of the three-electrode system and the reaction on the working electrode. Glucose diffuses through the analytic liquid and adsorbs on the working electrode. Then it is oxidized to gluconolactone by GOx immobilized on the ZnO-Ag, and meanwhile GOx is changed into GOx(FADH_2_). Afterwards, GOx(FADH_2_) is reverted to GOx through the reaction with O_2_ dissolved in the solution. During this process, H_2_O_2_ is produced and releases electrons. These generated electrons transfer from the redox active centers of GOx to ZnO-Ag and then to the working electrode. By means of detecting the electric signals, the concentration of glucose is determined.

Figure 6b shows that the Nafion/GOx/ZnO-Ag/ITO glucose sensors exhibit much higher response currents than the Nafion/GOx/ZnO/ITO one does when 100 μL glucose solution is successively added in 50 mL PBS. From the calibration curves of glucose concentration versus the response currents in Figure 6c, the performance parameters of the Nafion/GOx/ZnO/ITO, Nafion/GOx/ZnO-Ag-1/ITO, Nafion/GOx/ZnO-Ag-2/ITO, and Nafion/GOx/ZnO-Ag-3/ITO glucose sensors are acquired. The sensitivities are separately 0.75, 1.34, 3.85, and 2.85 μA/(mM·cm^2^). Based on the signal-to-noise ratio of 3, the detection limits are determined to be 2.1, 1.9, 1.5, and 1.4 μM. Obviously, doping with Ag increases the sensitivities by 1.8, 5.1, and 3.8 times, and decreases the detection limits by 0.9, 0.7, and 0.7 times. The larger sensitivities and lower detection limits of the Nafion/GOx/ZnO-Ag/ITO glucose sensors are mainly ascribed to the fast electron transfer rate of the ZnO-Ag nanorods as well as the better catalytic character of GOx. The linear ranges are 2.1 × 10^−3^–10.0, 1.9 × 10^−3^–6.0, 1.5 × 10^−3^–6.5, and 1.4 × 10^−3^–5.0 mM. Apparently, the upper limits of the Nafion/GOx/ZnO-Ag/ITO glucose sensors diminish. This is because the surface energy of the ZnO-Ag nanorods decreases, thus GOx easily falls off the ZnO-Ag nanorods into the analytic liquid [25]. The Michaelis-Menten constants of the Nafion/GOx/ZnO-Ag/ITO glucose sensors are calculated to be 6.17, 5.4, and 3.87 mM, which are smaller than that (6.49 mM) of the Nafion/GOx/ZnO/ITO glucose sensor. The lower Michaelis-Menten constants reveal that the ZnO-Ag nanorods provide a favorable environment for GOx and facilitate the electron transfer reaction.

In comparison to the Nafion/GOx/ZnO/ITO glucose sensor, the Nafion/GOx/ZnO-Ag/ITO glucose sensors exhibit better performance. This is ascribed to the combined effects of the doped Ag; the surface morphologies, wettability, and electron transfer capability of the ZnO-Ag nanorods; the contact area of solid-liquid interface; and the catalytic character of GOx on the performance of the glucose sensors. Of all the Nafion/GOx/ZnO-Ag/ITO glucose sensors, the Nafion/GOx/ZnO-Ag-2/ITO one shows the best performance, thus the optimal amount of doped Ag is determined to be 2 mM.

#### 3.3.4. Comparison of the Glucose Sensors in This and Previous Works

As shown in Table 1, the detection limits of the ZnO-Ag-based glucose sensors are slightly smaller than those reported in the literature [13,14,17], whilst the linear ranges are close to the corresponding values. Whereas the sensitivities of the ZnO-Ag-based glucose sensors approach that of the ZnO-Al-based one, they are much smaller than that of the ZnO-Ni- and ZnO-Co-based ones. This is attributed to the different electron transfer abilities or catalytic activities of Ag, Al, Ni, and Co. Nonetheless, this work focuses on the combined effects of the aforementioned influencing factors on the performance of glucose sensors. Therefore, more attention in this paper is paid to establishing the relationship and optimizing the amount of doped Ag.

#### 3.3.5. Selectivity and Storage Stability

**Figure 7:**
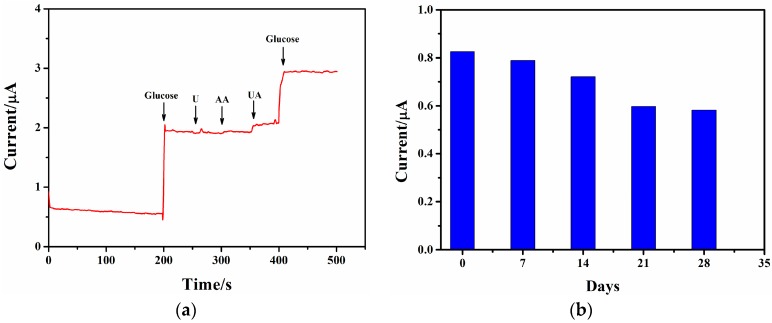
(**a**) The selectivity of the Nafion/GOx/ZnO-Ag-2/ITO glucose sensor upon the successive addition of 3 mM glucose, 0.5 mM urea (U), 0.1 mM ascorbic acid (AA), 0.5 mM uric acid (UA), and 3 mM glucose in PBS at +0.8V; (**b**) The storage stability of the glucose sensor measured in 1 mM glucose solution every seven days.

With the successive addition of 3 mM glucose, 0.5 mM U, 0.1 mM AA, 0.5 mM UA, and 3 mM glucose in PBS at +0.8 V, the selectivity of the Nafion/GOx/ZnO-Ag-2/ITO glucose sensor was investigated. Figure 7a shows that the currents increase significantly with glucose injected, but almost keep unchanged with U, AA, and UA added. This implies that the glucose sensor has excellent selectivity to glucose in the presence of interfering substances.

Figure 7b displays the current response of the Nafion/GOx/ZnO-Ag-2/ITO glucose sensor in 1 mM glucose solution every seven days. The oxidation currents respectively keep 95.5% and 87.3% of their initial values seven and 14 days later. This is primarily caused by the leakage of GOx from the ZnO-Ag nanorods to the analytical liquid. After 21 and 28 days, the oxidation currents remain at 71.6% and 70.5% of their initial values. All of the results suggest that the Nafion/GOx/ZnO-Ag-2/ITO glucose sensor has good storage stability. This is attributed to the better biocompatibility of the ZnO-Ag nanorods, which provides a favorable environment for GOx to retain its intrinsic bioactivity.

## 4. Conclusions

The performance of the ZnO nanorod-based enzymatic glucose sensor was improved with Ag doping in the ZnO nanorods, and was optimized by regulating the amount of doped Ag. The doped Ag remarkably facilitates the electron transfer rate of the ZnO-Ag nanorods and enhances the catalytic character of GOx, although it slightly diminishes the surface roughness of the ZnO-Ag nanorods, weakens its hydrophilicity, and further decreases the contact area of the solid-liquid interface between the electrode and analytic liquid. Therefore, the sensitivities of the ZnO-Ag nanorod-based glucose sensors with doped Ag of 1, 2, and 3mM increase by 1.8, 5.1, and 3.8 times, and the detection limits decrease by 0.9, 0.7, and 0.7 times in comparison to those of the ZnO nanorod-based glucose sensor. Additionally, the glucose sensor corresponding to the Ag dopant of 2 mM exhibits the optimal performance, with a sensitivity of 3.85 μA/(mM·cm^2^), detection limit of 1.5 μM, linear range of 1.5 × 10^−3^–6.5 mM, and Michaelis-Menten constant of 3.87 mM. Moreover, the optimal glucose sensor has good selectivity to urea, ascorbic acid, and uric acid, and shows excellent storage stability. The results indicate that ZnO-Ag nanorods are attractive materials for the fabrication of other enzymatic biosensors, such as urea, uric acid, cholesterol, and hydrogen peroxide sensors.

## Figures and Tables

**Table 1:** Comparison of the glucose sensors in this and previous works.

Nanostructures	Sensitivity μA/(mM·cm^2^)	Detection Limit μM	Linear Range mM	Reference
ZnO	0.75	2.1	2.1 × 10^−3^–10.0	This work
ZnO-Ag-1	1.34	1.9	1.9 × 10^−3^–6.0	This work
ZnO-Ag-2	3.85	1.5	1.5 × 10^−3^–6.5	This work
ZnO-Ag-3	2.85	1.4	1.4 × 10^−3^–5.0	This work
ZnO-Ni	61.78	2.5	0.5–8	[13]
ZnO-Co	13.3	20	0–4	[14]
ZnO-Al	5.5	167	0.28–28	[17]

## References

[1] Wang J. (2008). Electrochemical glucose biosensors. Chem. Rev..

[2] Khun K., Ibupoto Z.H., Lu J., Alsalhi M.S., Atif M., Ansari A.A., Willander M. (2012). Potentiometric glucose sensor based on the glucose oxidase immobilized iron ferrite magnetic particle/chitosan composite modified gold coated glass electrode. Sens. Actuators B Chem..

[3] Zhao Z., Lei W., Zhang X., Wang B., Jiang H. (2010). ZnO-based amperometric enzyme biosensors. Sensors.

[4] Wei A., Pan L., Huang W. (2011). Recent progress in the ZnO nanostructure-based sensors. Mater. Sci. Eng. B.

[5] Ahmad R., Tripathy N., Jin H.K., Hahn Y.B. (2012). Highly selective wide linear-range detecting glucose biosensors based on aspect-ratio controlled ZnO nanorods directly grown on electrodes. Sens. Actuators B Chem..

[6] Yang K., She G.W., Wang H., Ou X.M., Zhang X.H., Lee C.S., Lee S.T. (2009). ZnO nanotube arrays as biosensors for glucose. J. Phys. Chem. C.

[7] Wang J.X., Sun X.W., Wei A., Lei Y., Cai X.P., Li C.M., Dong Z.L. (2006). Zinc oxide nanocomb biosensor for glucose detection. Appl. Phys. Lett..

[8] Xu C.X., Sun X.W., Dong Z.L., Yu M.B. (2004). Zinc oxide nanodisk. Appl. Phys. Lett..

[9] Tereshchenko A., Bechelany M., Viter R., Khranovskyy V., Smyntyna V., Starodub N., Yakimova R. (2016). Optical biosensors based on ZnO nanostructures: advantages and perspectives. A review. Sens. Actuators B Chem..

[10] Fang L., Liu B., Liu L., Li Y., Huang K., Zhang Q. (2016). Direct electrochemistry of glucose oxidase immobilized on Au nanoparticles-functionalized 3D hierarchically ZnO nanostructures and its application to bioelectrochemical glucose sensor. Sens. Actuators B Chem..

[11] Tian K., Alex S., Siegel G., Tiwari A. (2015). Enzymatic glucose sensor based on Au nanoparticle and plant-like ZnO film modified electrode. Mater. Sci. Eng. C.

[12] Anusha J.R., Kim H.J., Fleming A.T., Das S.J., Yu K.H., Kim B.C., Raj C.J. (2014). Simple fabrication of ZnO/Pt/chitosan electrode for enzymatic glucose biosensor. Sens. Actuators B Chem..

[13] Chu X., Zhu X., Dong Y., Chen T., Ye M., Sun W. (2012). An amperometric glucose biosensor based on the immobilization of glucose oxidase on the platinum electrode modified with NiO doped ZnO nanorods. J. Electroanal. Chem..

[14] Zhao Z.W., Chen X.J., Tay B.K., Chen J.S., Han Z.J., Khor K.A. (2007). A novel amperometric biosensor based on ZnO:Co nanoclusters for biosensing glucose. Biosens. Bioelectron..

[15] Wei Y., Li Y., Liu X., Xian Y., Shi G., Jin L. (2010). ZnO nanorods/Au hybrid nanocomposites for glucose biosensor. Biosens. Bioelectron..

[16] Liu J., Guo C., Li C.M., Li Y., Chi Q., Huang X., Liao L., Yu T. (2009). Carbon-decorated ZnO nanowire array: A novel platform for direct electrochemistry of enzymes and biosensing applications. Electrochem. Commun..

[17] Fidal V.T.K.P., Inguva S., Krishnamurthy S., Marsili E., Mosnier J.P., Chandra T.S. (2017). Mediator-free interaction of glucose oxidase, as model enzyme for immobilization, with Al-doped and undoped ZnO thin films laser-deposited on polycarbonate supports. Enzyme Microb. Technol..

[18] Patil S.S., Mali M.G., Tamboli M.S., Patil D.R., Kulkarni M.V., Yoon H., Kim H., Al-Deyab S.S., Yoon S.S., Kolekar S.S. (2016). Green approach for hierarchical nanostructured Ag-ZnO and their photocatalytic performance under sunlight. Catal. Today.

[19] Zheng Y., Zheng L., Zhan Y., Lin X., Zheng Q., Wei K. (2007). Ag/ZnO heterostructure nanocrystals: Synthesis, characterization, and photocatalysis. Inorg. Chem..

[20] Xian F., Miao K., Bai X., Ji Y., Chen F., Li X. (2013). Characteraction of Ag-doped ZnO thin film synthesized by sol–gel method and its using in thin film solar cells. Optik.

[21] Mu-Hsiang H., Chi-Jung C. (2014). Ag-doped ZnO nanorods coated metal wire meshes as hierarchical photocatalysts with high visible-light driven photoactivity and photostability. J. Hazard. Mater..

[22] Zhou F., Jing W., Wu Q., Gao W., Jiang Z., Shi J., Cui Q.B. (2016). Effects of the surface morphologies of ZnO nanotube arrays on the performance of amperometric glucose sensors. Mater. Sci. Semicond. Process..

[23] Quéré D. (2008). Wetting and roughness. Annu. Rev. Mater. Res..

[24] Baszkin A., Lyman D.J. (1980). The interaction of plasma proteins with polymers. I. Relationship between polymer surface energy and protein adsorption/desorption. J. Biomed. Mater. Res..

[25] Michiardi A., Aparicio C., Ratner B.D., Planell J.A., Gil J. (2007). The influence of surface energy on competitive protein adsorption on oxidized NiTi surfaces. Biomaterials.

[26] Shi Y., Wen L., Li F., Cheng H.M. (2011). Nanosized Li_4_Ti_5_O_12_/graphene hybrid materials with low polarization for high rate lithium ion batteries. J. Power Sources.

